# Factors Influencing Seed Dormancy and Germination and Advances in Seed Priming Technology

**DOI:** 10.3390/plants13101319

**Published:** 2024-05-10

**Authors:** Yanfeng Fu, Li Ma, Juncai Li, Danping Hou, Bo Zeng, Like Zhang, Chunqing Liu, Qingyu Bi, Jinsong Tan, Xinqiao Yu, Junguo Bi, Lijun Luo

**Affiliations:** 1 Shanghai Agrobiological Gene Center, Shanghai 201106, China; fuyanfeng@webmail.hzau.edu.cn (Y.F.); yxq@sagc.org.cn (X.Y.); lijun@sagc.org.cn (L.L.); 2 Key Laboratory of Grain Crop Genetic Resources Evaluation and Utilization, Ministry of Agriculture and Rural Affairs, Shanghai 201106, China; ljc17549636237@163.com (J.L.); hdp@sagc.org.cn (D.H.); bqy@sagc.org.cn (Q.B.); tjs@sagc.org.cn (J.T.); 3 National Key Laboratory of Crop Genetic Improvement, College of Plant Science and Technology, Huazhong Agricultural University, Wuhan 430070, China; 4 Institute for Sustainable Horticulture, Kwantlen Polytechnic University, 20901 Langley Bypass, Langley, BC V3A 8G9, Canada; li.ma6@kpu.ca; 5 National Agricultural Technology Extension Service Center, Room 622, Building 20, Maizidian Street, Chaoyang District, Beijing 100125, China; zengbo@agri.gov.cn (B.Z.); zhanglike@agri.gov.cn (L.Z.); liuchunqing@agri.gov.cn (C.L.)

**Keywords:** seed physiology, seed dormancy, germination, phytohormone, seed priming

## Abstract

Seed dormancy and germination play pivotal roles in the agronomic traits of plants, and the degree of dormancy intuitively affects the yield and quality of crops in agricultural production. Seed priming is a pre-sowing seed treatment that enhances and accelerates germination, leading to improved seedling establishment. Seed priming technologies, which are designed to partially activate germination, while preventing full seed germination, have exerted a profound impact on agricultural production. Conventional seed priming relies on external priming agents, which often yield unstable results. What works for one variety might not be effective for another. Therefore, it is necessary to explore the internal factors within the metabolic pathways that influence seed physiology and germination. This review unveils the underlying mechanisms of seed metabolism and germination, the factors affecting seed dormancy and germination, as well as the current seed priming technologies that can result in stable and better germination.

## 1. Introduction

**Figure 1:**
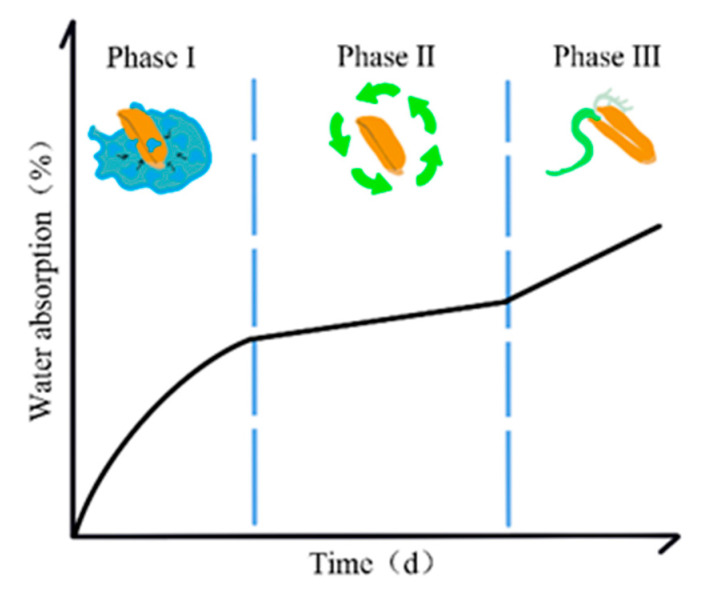
Three phases of seed germination. Phase I (imbibition), phase II (sluggish imbibition), phase III (germination).

Seeds serve as the starting point of a plant’s vegetative growth. In seeds, important developmental processes take place that are necessary for the growth and development of embryos into plumules and radicles, and ultimately result in the emergence of a new plant [1]. Seed dormancy, one of the least understood phenomena in seed biology [2], is an adaptive trait acquired by plants during extended periods of phylogenetic evolution. It plays an important role in shaping the morphological development of plants from seedling emergence to maturity, and it preserves the offspring by preventing losses caused by seed germination at unsuitable times. Seed germination is a critical phase in the initial plantlet development, which is governed by various physiological and environmental factors. This process is an orderly series of physiological activities and morphogenesis, which starts from physical water absorption called imbibition (Figure 1) [3]. These processes hold significant implications for effective field management, affecting both the yield and crop quality.

However, deeply dormant seeds often encounter delayed germination and abnormal seedlings, which pose a significant challenge in various academic fields, including breeding, agroforestry, and agricultural production. Seed dormancy was classified into three categories as follows: eco-dormancy, regulated by environmental factors; para-dormancy, regulated by physiological factors outside of the affected structure; and endo-dormancy, controlled by physiological factors inside of the affected structure [4]. Baskin and Baskin further classified seed dormancy into five types as follows: physiological, morphological, morpho-physiological, physical, and combinational dormancy [5]. Although the causes of these various types of dormancy may vary, the physiological mechanisms underlying the transition from a dormant to a germinated state should remain consistent once the seeds have met the required conditions for germination.

Seed priming is a technique pioneered by Heydecker to regulate slow water absorption in seeds and subsequently control the drying process [6]. This process starts with soaking seeds in a treatment solution to slowly absorb water, which is followed by dehydrating the swollen seeds back to their initial state. A series of physiological and biochemical changes occur within the seeds throughout this process [7]. This technology employs physical, chemical, or biological treatments to enhance the seed quality, maintain the seeds in Phase II without triggering complete germination, and activate several seed restoration responses, such as DNA repair and peroxide scavenging, to enable better seed germination, particularly when exposed to biotic or abiotic stresses.

The widely investigated mechanisms of seed dormancy and germination play an essential role in seeds. Existing studies on this aspect fall into two main categories as follows: understanding the mechanisms of the transition from dormancy to germination, and exploring the methods used to artificially break dormancy and effectively control the germination process. While the former category mainly focuses on metabolic pathways, such as various plant hormones and proteins within their respective signaling pathways, the latter, exemplified by seed priming technology, has recently gained increasing attention. In this review, we summarize the mechanisms underlying the regulation of seed dormancy and germination, with a particular focus on the phytohormone signaling network. We provide an overview of these seed priming technologies and their related research advances.

## 2. Factors Influencing Seed Dormancy and Germination

### 2.1. Seed Structure

**Figure 2:**
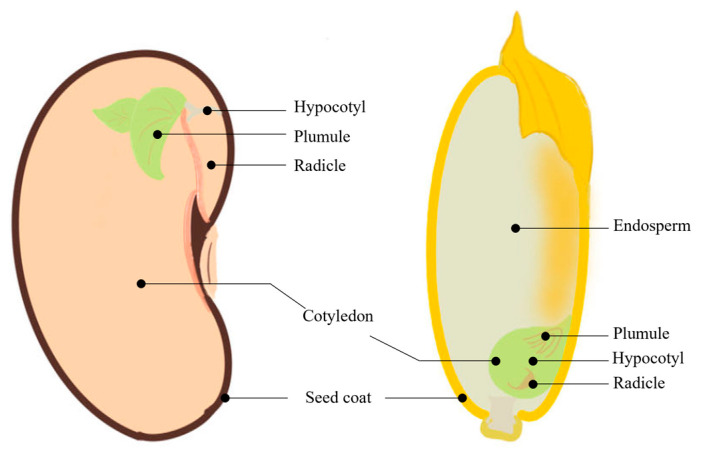
Seed structure diagram. Dicotyledonous plants, exemplified by a kidney bean seed (**left**, no endosperm), and monocotyledonous plants, exemplified by a rice seed (**right**, with endosperm).

The seeds of monocotyledonous plants, such as cereal seeds like rice and wheat, have a distinctive structure, consisting of an embryo, a seed coat, and an endosperm [8]; the embryo comprises four primary components as follows: the radicle, hypocotyl, plumule, and cotyledon. In contrast, the seeds of dicotyledonous plants, such as legumes like soybeans and broad beans, consist of only an embryo and seed coat, lacking an endosperm (Figure 2). Some dicots such as Arabidopsis and 60% of legumes have a thin layer of endosperm left. In Arabidopsis, this is an important temperature sensor, preventing germination at high temperatures [9]. The structural composition of these seeds acts as a barrier to water uptake; specifically, the seed coat and shell restrict the entry of both oxygen and water, inhibiting seed germination and limiting embryo growth. It has been shown that the poor permeability of the seed coat is a key contributor to seed dormancy [10].

Under favorable conditions, dry seeds gradually resume their metabolic activity by absorbing water from the environment, thus enabling them to complete essential cellular activities in preparation for subsequent seedling growth. Upon saturation with water, the radicle continues to elongate until it breaks through the seed coat and eventually bursts out of the hilum. Following the radicle’s breakthrough, the hypocotyl begins to elongate and then the embryo grows [11]. The emergence of the radicle from the seed coat signifies the completion of the seed germination process.

### 2.2. Nutrient Change 

Seeds consume their own stored substances to provide the energy needed for germination. In mature dry seeds, many of the physiological and biochemical processes involving enzymes do not take place due to the absence of suitable conditions or the lack of key components. Once the conditions required for the reaction have been reached, with the combined action of these enzymes, large storage substances, such as starch, fat, and protein, are converted into sugar via oxidative respiration, making them transportable and utilizable. Throughout dormancy, the seeds do not perform physiological activities, and the reactivation of growth at the end of the dormant period requires an influx of energy, which is provided by sugar [12].

#### 2.2.1. Starch

Starch comprises amylose and amylopectin, both having linear and branched molecules of the α-1,4-glucosidic type, with α-1,6-linkages at the branching points [13]. Adenosine 5′-diphophate glucose (ADP-glucose), a precursor substance for starch synthesis, is synthesized by ADP-glucose pyrophosphorylase (AGPase, E.C.2.7.7.27). Starch is typically produced and deposited in storage cells, in the cotyledons, or in persistent endosperm, serving as long-term carbon sources to support eventual seedling establishment [14].

During seed germination, starch is hydrolyzed by amylase to generate micromolecular dextrin, which is further hydrolyzed to generate maltose, and ultimately maltase is hydrolyzed to produce glucose, which is used as the energy source for plumule and radicle growth. Depending on the type of the isomerization of the enzyme hydrolysis product, these amylases can be classified as α-amylase (E.C.3.2.1.1), β-amylase (E.C.3.2.1.2), γ-amylase (E.C.3.2.1.3), and amylo-α-1,6-glucosidase (E.C.3.2.1.33). Other studies have shown that germinating seeds are more physiologically and metabolically active than ungerminated seeds, with the starch content decreasing and the total and reducing sugar contents increasing during germination [15,16,17]. Sugars play a crucial role in seed germination, as they provide energy through the breakdown of glucose via glycolysis and other sugar metabolism pathways.

#### 2.2.2. Fat

Fat, the primary energy reserve of oil seeds, is abundant in legume seeds in the form of triacylglycerols. During the initial oilseed maturation, large amounts of free fatty acids are produced, which are mainly used to synthesize fat. Moreover, the synthesis of fat during seed maturation progresses after the conversion of saturated fatty acids to unsaturated fatty acids, initially catalyzed by fatty acid desaturase, and eventually resulting in synthesis.

During germination, triacylglycerols are degraded by lipase into glycerol and free fatty acids, promoting seedling growth. These free fatty acids are subsequently broken down and ultimately converted to sugars as substrates for respiration through gluconeogenesis [18]. Fat is a non-polar compound stored in an anhydrous state, whereas glycogen, the energy storage site, is a polar compound stored in a hydrated state. The metabolizable energy of fat is six times higher than that of glycogen of the same weight. Fatty acid catabolism occurs in the mitochondrial matrix, where fatty acids are first activated with coenzyme-A by fatty acid thiokinase 1, thus enabling their entry into the mitochondria for β-oxidation. This process results in the formation of acetyl-CoA, subsequently generating energy through the tricarboxylic acid cycle.

#### 2.2.3. Protein

Proteins play an indispensable role in the essential functions of cells and living organisms. During germination, the stored proteins are hydrolyzed by proteases, releasing amino acids and small molecule peptides. Amino acids can provide energy through the oxidation of the carbon skeleton after deamination or as building blocks for the synthesis of new proteins [19]. In addition, amino acids can also be converted into common metabolic intermediates, such as pyruvate, oxaloacetate, and α-ketoglutarate. In essence, they are the “fuel” for various metabolic processes within an organism.

Protein phosphorylation modifications also are crucial in regulating seed dormancy and germination. Currently, the four following primary kinases have been extensively investigated in seed dormancy and germination: mitogen-activated (MAPKs), calcium-dependent (CDPKs), sucrose non-fermentation 1-related (SnRKs), and receptor-like protein kinases (RLKs) [20]. These kinases primarily regulate dormancy and germination by responding to signaling cues from phytohormones.

### 2.3. Endogenous Hormones Signaling and Gene Regulation

Phytohormones are vital for the regulation of seed dormancy and germination. According to the doctrine of endogenous hormone regulation proposed by Khan in 1975, the interactions among gibberellin acid (GA), cytokinin (CTK), and abscisic acid (ABA) determine seed dormancy and germination. For instance, when both ABA and GA are present within seeds, the germination-promoting effect of GA is inhibited by ABA; when GA, CTK, and ABA are simultaneously present, CTK exerts an anti-inhibitory effect, facilitating germination [21]. The antagonistic effects between ABA and GA in the regulation of seed dormancy and germination have been demonstrated in rice and tobacco [22,23]. Additionally, the other endogenous hormones, such as ethylene (ETH), brassinolide (BR), auxin, and strigolactones (SLs), are also involved in regulating the physiological and biochemical processes of seed dormancy and germination [24,25,26]. To further investigate the regulatory mechanisms of endogenous hormones during seed dormancy and germination, some researchers conducted a detailed analysis of the relevant genetic locus [27,28].

#### 2.3.1. Abscisic Acid

ABA is an essential phytohormone in regulating dormancy. The induction of dormancy during seed development is closely related to the elevation of the level of endogenous ABA in plants, as demonstrated in other studies concerning Arabidopsis and tomatoes [29]. 

There are four types of ABA binding proteins in Arabidopsis, namely Flowering Time Control Locus A (FCA), ABA receptor (ABAR), G-protein Coupled Receptor (GPCR, 7 transmembrane receptor), and PYRABACTIN RESISTANCE (PYR)/PYR1-LIKE (PYL)/REGULATORY COMPONENT OF ABA RECEPTOR (RCAR). Among these, FCA functions as an RNA-binding protein that controls the flowering time. ABAR, similar to the bean protein Mg-chelatase H subunit (ChlH), produces Mg-protoporphyrin Ⅸ (Mg-proto) which coordinates nuclear and chloroplast gene expression. This coordination, in turn, elicits the response of the downstream component ABSCISIC ACID INSENSITIVE4 (ABI4) [30,31,32]. 

**Figure 3:**
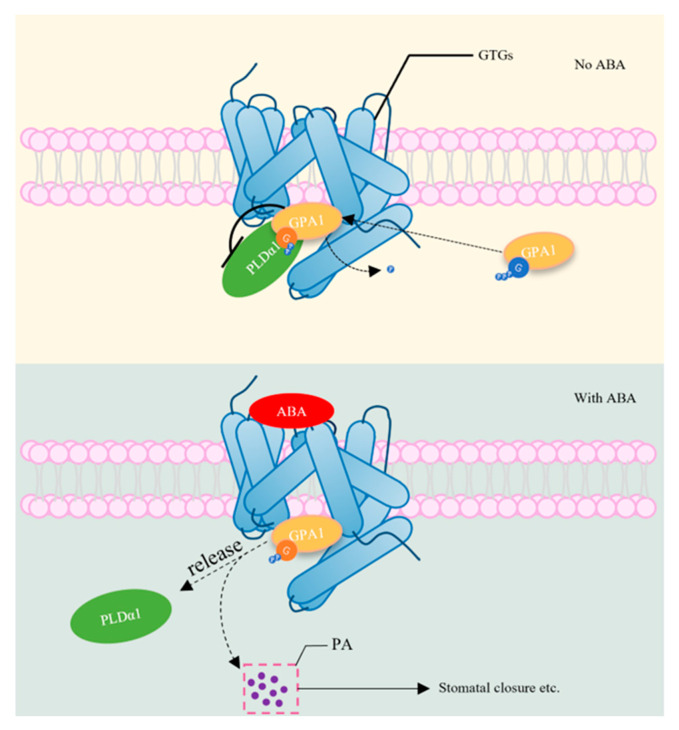
GTGs regulate the ABA signaling pathway. GPCRs are a family of receptors with a seven-transmembrane helix (7TM) structure on the cell membrane. GPCR-type G proteins (GTGs) can bind the GTP-bound form of GTPase-accelerating protein 1 (GAP1), render the dephosphorylation of GTP-GAP1, and allow GDP-GAP1 to bind to and inhibit PLDα1. When ABA is present, PLDα1 is released, producing phosphatidic acid (PA) that induces stomatal closure and a series of physical processes [34].

The regulatory mechanisms of GPCR and PYR/PYL/RCAR have been well studied and will be discussed in detail to shed light on these pathways. GPCR-type *G* (GTG) proteins, specifically GTG1 and GTG2, are topologically similar to GPCRs in Arabidopsis, featuring nucleotide-binding and GTPase-activating domains that differentiate them from the other GPCRs. In the ABA signaling pathway, the GTGs coordinate with the GTPase-accelerating protein (GPA)1, which is encoded by the sole Arabidopsis G-alpha subunit gene (*GPA1*), to regulate various physiological processes. The GTP-bound form of *GPA1* predominates in vivo and negatively regulates the GTPase activity of the GTGs [33]. The GDP-bound form of the GTGs represents a high-affinity-binding state, facilitating the binding of ABA to GTGs. GDP-bound *GPA1* can engage with and inhibit the phospholipase PLDα1. When ABA signaling is initiated, PLDα1 is released, resulting in the production of phosphatidic acid, which promotes ABA-induced stomatal closure, gene expression, and other stress responses, as illustrated in Figure 3 [34]. 

When the concentration of ABA increases, members of the PYR/PYL/RCAR protein family detect ABA signaling, leading to the suppression of type 2C PROTEIN PHOSPHATASE (PP2Cs) coreceptors, which results in the release of SUCROSE NONFERMENTING1-RELATED KINASE2 (SnRK2) from the PP2C-SnRK2 complexes [31,35,36]. Subsequently, SnRK2 catalyzes the phosphorylation of the ABA-responsive element binding protein/ABA-responsive element binding factor (AREB/ABF), which regulates the expression of downstream response genes [35]. Previous studies have identified various transcriptional components involved in ABA signaling transduction. Notably, the ABSCISIC ACID INSENSITIVE5 (ABI5) transcription factor, which is a member of the basic leucine zipper (bZIP) family, exhibits a robust response to ABA [37]. ABI5, together with ABSCISIC ACID INSENSITIVE3 (ABI3) and ABI4, are key components in seed-specific ABA signaling.

#### 2.3.2. Auxins

Auxins function as an inhibitor of seed sprouting in many geophytes. In the case of Arabidopsis, auxin plays a pivotal role in both promoting dormancy and inhibiting germination by intensifying ABA signaling [38]. In general, phytohormones work synergistically to maintain seed dormancy. The molecular mechanisms underlying these processes are discussed below.

**Figure 4:**
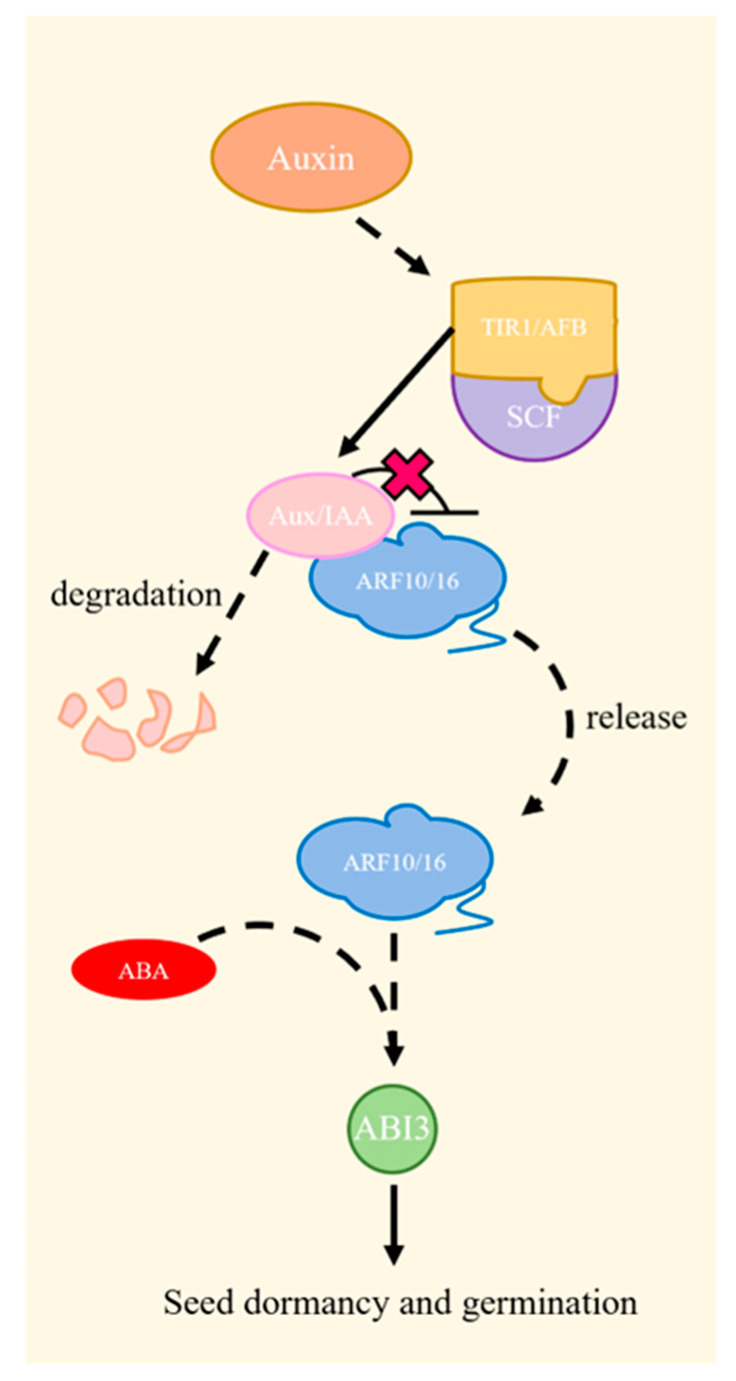
A schematic of the auxin signaling pathway. When auxins are present, Aux/IAA proteins are degraded by the SCF^TIR1/AFB^ complex-26S proteasome after TIR1/AFB detects auxins signaling, allowing ARF10/16 to work with ABA to regulate seed dormancy and germination via ABI3 [38].

Auxin regulation involves F-box proteins, specifically TRANSPORT INHIBITOR RESPONSE1 (TIR1)/AUXIN SIGNALING F-BOX (AFB), auxin/indole-3-acetic (Aux/IAA) proteins, and AUXIN RESPONSE FACTOR (ARF). Upon the perception of auxin signaling by TIR1/AFB, Aux/IAA is recruited for degradation via the SCF^TIR1/AFB^ complex-26S proteasome pathway, which alleviates the inhibition of ARF transcription factors in order to elicit or silence the expression of downstream auxin-responsive genes [39,40,41]. Previous research has revealed a reciprocal relationship between auxin signaling and the ABA signaling pathways in seed dormancy. ARF10 and ARF16, major factors of auxins, are recruited to regulate the expression of ABI3 during seed dormancy, as illustrated in Figure 4 [38].

#### 2.3.3. Gibberellins Acid (GAs)

GAs are multifunctional physiological regulators involved in various aspects of plant development, such as shoot elongation, root development, flowering, and seed germination. Endogenous GAs have been observed to promote radicle protrusion, thereby accelerating seed germination [42,43,44,45]. In wheat and rice seeds, GAs are the primary phytohormones regulating dormancy, where the balance of ABA/GAs is critical to a dormant degree, with a higher GA concentration resulting in less dormancy [46,47].

**Figure 5:**
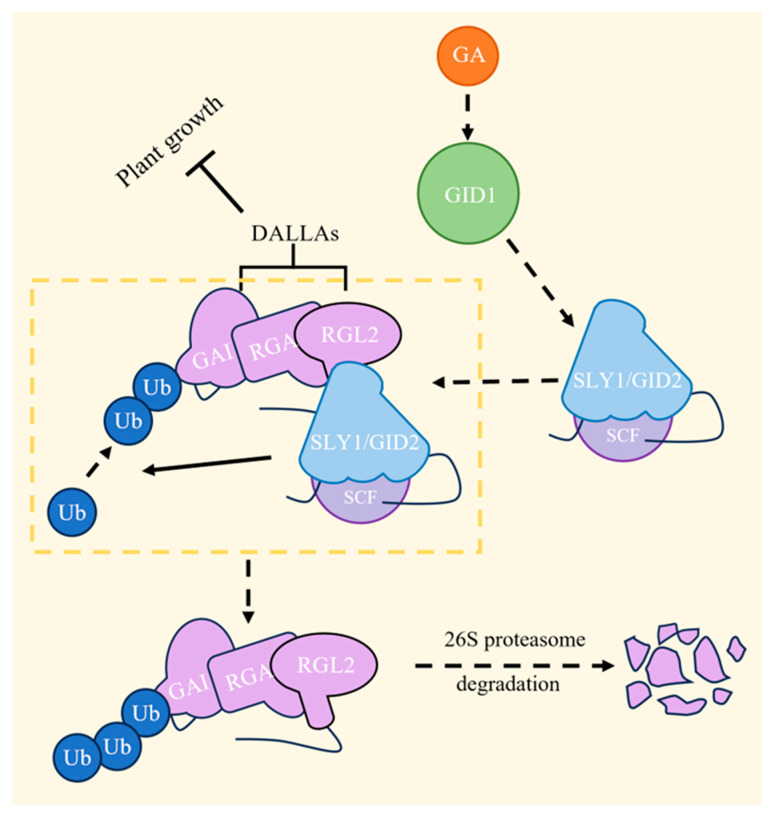
GA signaling pathway. GA binds to GIBBERELLIN-INSENSITIVE DWARF1 (GID1), then DELLA growth inhibitors (DELLAs) are recruited to be ubiquitinated by SCF SLEEPY1 (SLY1)/GID2, and, ultimately, the ubiquitinated DELLAs are degraded by the 26S proteasome [48].

Upon the perception of GA by the nuclear receptor GIBBERELLIN-INSENSITIVE DWARF1 (GID1), the GA-GID1 complex recruits DELLA growth inhibitors (DELLAs include GAI, RGA, and RGL2), members of the GRAS family, which function as repressors of plant growth. Subsequently, the SCF SLEEPY1 (SLY1)/GID2 complex ubiquitinates DELLAs, marking them for degradation in the presence of 26S proteasome (Figure 5) [48]. Moreover, GA regulation is affected by temperature. For example, SOMNUS (SOM) encodes a CCCH-Type Zinc Finger Protein [49] during regulation. GA deactivates *SOM* and promotes seed germination at low temperatures; however, at high temperatures, *SOM* expression is enhanced by DELLAs and ABA and epigenetically by the AGAMOUS-LIKE 67-EARLY BOLTING IN SHORT DAYS (AGL67-EBS) complex, inhibiting GA synthesis and preventing seed germination [50,51]. At ultra-high temperatures (above 32 °C), the interactions between the HEAT SHOCK PROTEIN (HSP) and HEAT SHOCK FACTOR (HSF) activate FUSCA3 (FUS3) protein synthesis and accumulation, triggering ABA synthesis and GA degradation, thereby impeding seed germination [52]. 

#### 2.3.4. Cytokinins (CTKs)

The effect of CTKs, which are widely recognized plant hormones that play a crucial role in promoting seed germination, varies across different varieties and is less potent than that of GA [53]. 

The discovery of CTKs as plant hormones with cell division-promoting properties dates back to 1955, when Miller and Skoog investigated the growth-promoting effects of autoclaved herring sperm DNA on tobacco callus tissue [54]. The CTK signal transduction pathway bears similarity to the His-Asp phosphorelay that was found in bacterial two-compound signaling systems [55,56]. This regulatory process involves a His residue in the sensor kinase and an Asp residue in the receiver domain. In plants, the CTK pathway is regulated by four alternating, sequential phosphorylation events, which include a His kinase, an Asp receiver domain, Arabidopsis histidine phosphotransfer proteins (AHPs), and separate response regulators (RRs), where the His kinase and the Asp receiver domain together form a ‘hybrid’ histidine kinase (HK) receptor [57,58]. 

**Figure 6:**
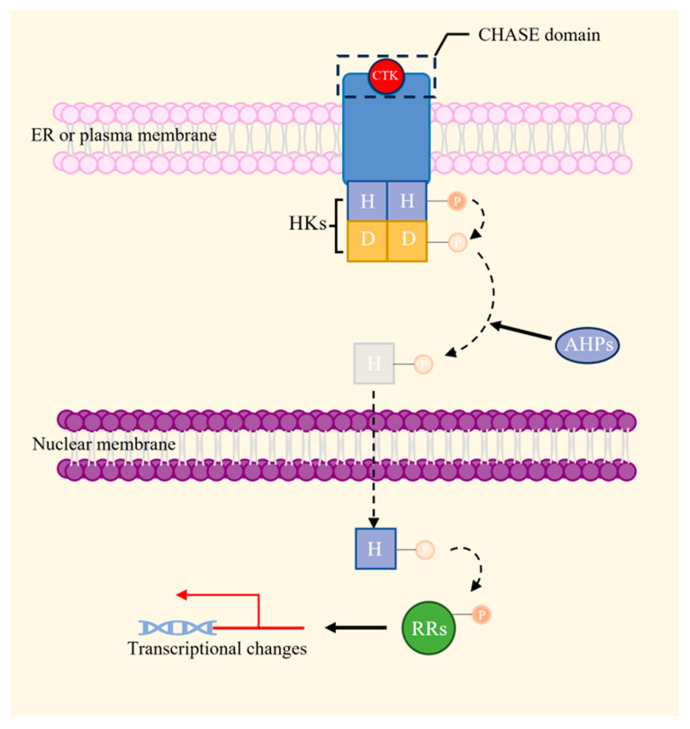
The schematic of CTKs signaling pathway. The CHASE domain of HK receptor binds CTKs and induces the histone-kinase domain phosphorylation. This is followed by multiple phosphoryl transfers, including from His to Asp, from Asp to radical His in the presence of AHPs, and from radical His to RRs in the nucleus, ultimately triggering transcriptional changes. The dotted line arrows indicate the transfer of substances, and the thick arrows indicate the facilitation of processes [66].

HK receptor features a conserved cytokinin-binding extracytosolic CHASE (cyclases/histidine kinase-associated sensing extracellular) domain, at least two transmembrane domains, a cytosolic region containing a histidine kinase domain, a canonical receiver domain, and a diverged receiver domain with an unlikely functionality [59,60,61]. This structural arrangement enables cytokinin binding to the CHASE domain to activate the cytosolic histone-kinase domain. This activation results in autophosphorylation on the conserved His residue, followed by the transfer of the phosphate group to a conserved Asp within the receiver domain [59,62,63,64]. This phosphorylation event is subsequently relayed to the downstream AHP and RR proteins, establishing a positive regulatory circuit. Ultimately, the cytokinin signal triggers a transcriptional change in the nucleus, as depicted in Figure 6 [65,66].

#### 2.3.5. Ethylene (ETH)

ETH, a gaseous phytohormone, dose-dependently alleviates seed dormancy and promotes germination. The exogenous application of ETH was found to significantly promote the germination of cotton and *Fraxinus mandshurica* seeds under osmotic stress [67,68], suggesting that ETH may function as a positive regulator in releasing seed dormancy, which is consistent with Kucera’s descriptions [69]. In contrast, elevated concentrations of ETH were found to not only delay seed germination, but to also inhibit radicle growth in pepper seeds [70]. This inhibition of seed germination by ETH has also been observed in *Camellia Oleifera* seeds [71]. This paradoxical effect of ETH necessitates a thorough understanding of its mechanisms of action particularly important.

The biosynthesis of ETH follows a well-established pathway. Initially, methionine (Met) is catalyzed by methionine adenosyl transferase (MAT) to yield S-adenosyl methionine (SAM); SAM is then converted into 1-aminocyclopropane-1-carboxylic acid (ACC) through ACC synthase (ACS). The subsequent oxidation of ACC by ACC oxidase (ACO) produces ETH, involving a circular reaction known as the Yang cycle [72]. In Arabidopsis, five membrane-localized ETH receptors have been identified as follows: ethylene response 1 (ETR1), ethylene response 2 (ETR2), ethylene resistant 1 (ERS1), ethylene resistant 2 (ERS2), and ethylene insensitive 4 (EIN4) [73,74,75,76,77,78]. The binding of ETH to its receptors, such as ETR1, deactivates the constitutive triple response 1 (CTR1) protein kinase via the HK domain of ETR1. Conversely, this process activates the kinase cascade that regulates ethylene insensitive 2 (EIN2) and its intranuclear transcription factors, including ethylene insensitive 3 (EIN3), EIN3-LIKE (EILs), and ethylene response element binding proteins (EREBPs)/ethylene responsive factors (ERFs), ultimately leading to the transcription of ethylene response genes [79]. 

**Figure 7:**
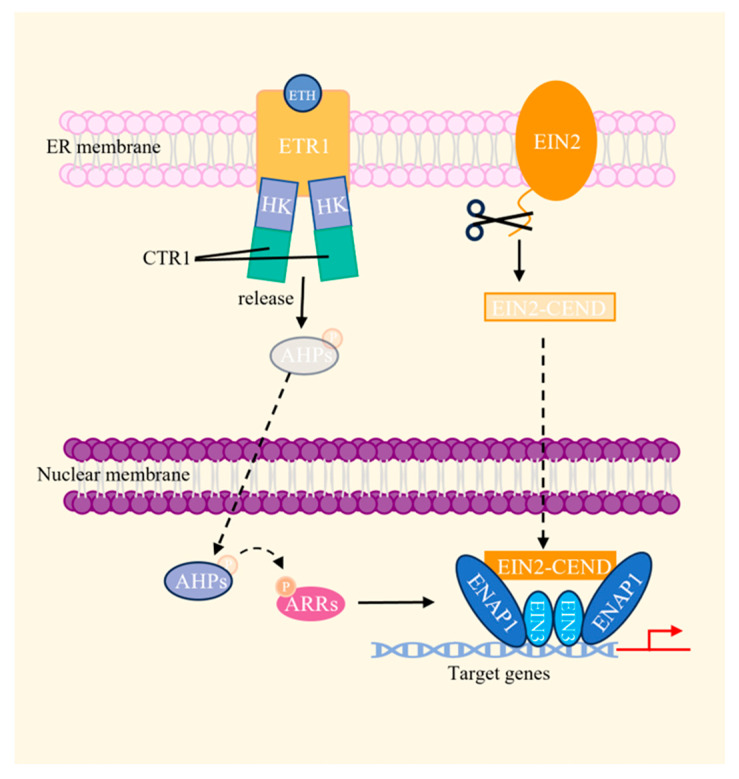
A schematic diagram of the ethylene (ETH) signaling pathway. The ETH receptor, ETR1, binds ETH and fails to activate CTR1, leading to the release of phosphate-carrying Arabidopsis histidine phosphotransfer proteins (AHPs). Simultaneously, the ethylene insensitive 2 (EIN2) undergoes degradation and the C-terminus is cleaved to produce EIN2-CEND. Both the AHPs and EIN2-CEND are transferred to the nucleus; the transfer of the phosphate groups on AHPs to ARRs results in transcriptional changes in the ethylene-responsive genes. EIN2-CEND interacts with the histone-binding protein EIN2 NUCLEAR-ASSOCIATED PROTEIN 1 (ENAP1) to enhance the ability of dimerized EIN3 to bind to the target gene and regulate EIN3-dependent transcriptional activation. The dotted line arrows indicate the transfer of substances, and thick arrows indicate the facilitation of these processes [80].

The recent advancements in ETH signaling pathway research include insights into the modified response elements and nuclear biochemical reactions [80]. The binding of ETH to its receptors, such as ETR1, results in the release of AHPs, which, in turn, leads to the dephosphorylation and cleavage of EIN2 to generate EIN2-CEND. Subsequently, AHPs and EIN2-CEND translocate to the nucleus, where the AHPs mediate the phosphoryl transfer to Arabidopsis response regulators (ARRs), ultimately participating in the final transcriptional processes. Additionally, EIN2-CEND interacts with the histone-binding protein EIN2 NUCLEAR-ASSOCIATED PROTEIN 1 (ENAP1), resulting in histone acetylation, which enhances the ability of dimerized EIN3 to bind to the target gene and regulate transcriptional activation (Figure 7) [81,82,83,84].

The significant promotion of seed germination and root and shoot growth, due to appropriate concentrations of brassinosteroids (BRs), was observed in tomato studies [85]. It has been shown that the mature seed transcriptome of Arabidopsis is highly temperature-sensitive; a low temperature during seed ripening induced the expression of several genes associated with dormancy, including the DELAY OF GERMINATION1 (DOG1) and C-REPEAT BINDING FACTORS (CBFs) [80]. These genes regulate seed dormancy by influencing the metabolism levels of GA and ABA within the seeds, thus modulating the dormancy state. The transcription rate of CBFs decreased at low temperatures and diminished in swollen seeds, indicating that the repression of CBF expression is a key feature of cold temperatures, promoting rather than inhibiting germination [86]. In this study, it was found that (a) seeds matured at low temperatures exhibited enhanced seed dormancy, correlating with the increased expression of DOG1; (b) there were significant geographic differences in DOG1 expression, with the southern seeds exhibiting significantly higher expression levels of DOG1 when compared to their northern counterparts at both the early and late developmental stages; and (c) when exposed to high temperatures, northern seeds exhibited higher germination rates than the southern seeds due to the high expression level of the DOG1 gene in the former, but there was no such difference between the seeds that matured under low-temperature conditions. In summary, DOG1 is a valuable tool to predict seed germination [87]. It has been revealed that the temperature fluctuations experienced within the maternal life history of Arabidopsis can trigger a signal transduction to the florigen factor flowering locus T (FT), which regulates seed dormancy by inhibiting the synthesis of pro-anthocyanidins in the fruit. This suggests that the periodicities determined by the temperature history of the maternal generation can affect the germination of offspring seeds by activating FT in the fruit [88].

The bioinformatic analysis of the genome sequence of Heading date 3a (Hd3a), homologous to Arabidopsis florigen gene FT, revealed the presence of an ABA-Responsive Element (ABRE), a response element in response to the ABA signaling. Comparative experiments confirmed that Hd3a can regulate rice seed germination in response to ABA signaling [89]. In addition, members of transcription factor series, such as AUX/IAA, basic helix–loop–helix (bHLH), and WRKY, play significant roles in the germination of common wild rice (*Oryz arufipogon* Griff.) seeds at different phases as follows: at the beginning of Phase I, at the end of Phase II, and during the transition from Phase I to Phase II, respectively. The temporal specificities of the gene expression of some transcription factor families reflect interspecific variations [3].

## 3. Seed Priming Technologies

Seed priming is the artificial treatment of seeds with natural or synthetic substances, which enables them to reach a specific physiological state prior to germination [90]. Primed seeds have twofold benefits as follows: enhanced and uniform emergence, and better yields [91]. Various approaches to seed priming include hydropriming, solid matrix, nano-priming, bio-priming, phytohormone priming, chemical priming, etc. Several widely applied and extensively researched methods are described below.

### 3.1. Hydropriming

Hydropriming is a technique in which the plant seeds are presoaked in water at an optimal temperature for a specified duration, followed by a natural drying process that returns them to their initial seed weight [90].

This method, characterized by its simplicity, cost-effectiveness, and practicality, is a viable technology that enhances the physiology metabolism within seeds, thereby improving their vigor. Hydropriming, along with other solvent-based priming operations that use water as a medium, involves immersing the seeds in a solution with a seed weight/solution volume ratio of 1:5 (*w*/*v*) [92]. The priming time and temperature vary depending on the seed variety; for example, the rice seed priming is usually conducted for 24 h at 25 °C in the dark [92]. Sunflower seeds subjected to hydropriming at 25 °C for 18 h showed a significantly better germination rate (92%), germination time, vigor index, and seedling dry weight (21 mg) when compared to the control [93]. Other research also showed that the seeds subjected to hydropriming exhibit higher rates of water imbibition, enhanced germination percentages, and increased seedling vigor [94,95]. While hydropriming can promote germination and seedling growth, it is crucial to note that this method can create favorable conditions for fungal contamination.

### 3.2. Solid Matrix Priming (SMP)

The American company Kamterter pioneered solid matrix priming for commercial seed priming in 1989 [96]. This technology precisely controls the water absorption rate through a solid matrix to facilitate seed priming. The seeds, solid substrate, and water are the three essential components. Dry seeds absorb water from the solid phase carrier until reaching equilibrium, emphasizing the pivotal role played by the physicochemical properties of the solid matrix within the solid matrix priming system. It has been shown that the seeds subjected to SMP exhibit a higher germination rate, a shorter germination time, and lower electrical conductivity values [97]. Furthermore, SMP media with other components, such as an Na-based hydrogel (sodium polyacrylate) and O_2_, were found to yield more effective results, particularly pronounced in lower-quality seeds [98,99].

The solid matrix used in the priming operation should have the following characteristics: high water-holding capacity, non-toxic effect, high permeability, low water solubility, chemically stable, low weight capacity, and easy separation from the seed after priming. Currently, commonly used solid matrixes include vermiculite, schist, diatomaceous soil, porous raw clay, sodium polypropionate gum, sand, soft bituminous coal, calcareous clay, shale, and synthetic calcium silicate [100]. Different SMPs were all best treated by vermiculite with an 80% water content at 25 °C for 4 days, which significantly improved seed germination and displayed the best priming effect [101].

### 3.3. Nano-Priming 

Nano-priming is a new technology that employs nanomaterials to enhance various facets of seed processing, handling, and quality [102]. Nanomaterials, due to their unique physiochemical properties, can change the arrangement and energy state of water molecules, thereby elevating cell membrane permeability and facilitating water absorption. Numerous studies show that nanomaterial priming can improve the germination rate of crop seeds and promote dry matter accumulation in seedlings [103,104,105,106,107]. For example, seed priming with the nanoparticles of micronutrients can improve both seed germination and seedling development. Additionally, the deployment of nanomaterials with reactive oxygen species-scavenging capabilities significantly enhances the plant performance under various abiotic stresses [108].

The procedure for nano-priming is similar to that of hydropriming. Most of the nanomaterials used for seed priming are mineral elements, such as cobalt (Co), zinc (Zn) and iron (Fe). It has been shown that metal-based nanomaterials (Co, Mn, Cu, Fe, Zn, Mo, and Se) can significantly improve pea seed germination performance and, thus, its field quality performance [109]. While nano-priming can significantly improve seed germination and seedling performances, particularly when under stress, it may also have some negative effects, such as the phytotoxicity. Therefore, it is urgent to address the issue of the metal toxicity of nano-priming to maintain the optimal seed priming effect [110].

### 3.4. Bio-Priming

Bio-priming is a seed treatment technique that involves pre-soaking and simultaneous inoculation with beneficial microorganisms. It is the seed pre-soaking along with the inoculation of beneficial microorganisms. Beneficial microorganisms or plant growth-promoting microorganisms used for seed treatment are crucial for biopriming, including mainly pseudomonas, enterobacter, xylaria, and bacillus. The inoculated microorganisms can colonize the inter-root surface of the plant and sustain the physiological growth of the plant for a long period; thus, bio-priming promotes crop maturity [111]. As other priming methods, bio-priming also intensifies the rate and homogeneity of seed germination, but also protects seeds against the soil and seed-borne pathogens. The hydration of seeds infected with pathogens during priming can result in a stronger microbial growth and subsequently weakening of plant vigor. Pea seedlings bio-primed with *Trichoderma asperellum* for 24 h showed increases in shoot length, root length, the no. of leaves, shoot fresh weight, root fresh weight, shoot dry weight, and root dry weight by 35.29, 96.49, 28.13, 36.10, 146.26, 30.17, and 77.2%, respectively [112]. Bio-primed maize seeds with *Azospirillum* displayed significantly higher field emergence (96.3%), crop growth, performance, and yield when compared to the control [113].

### 3.5. Phytohormone Priming

As the name implies, phytohormone priming entails submerging the seeds in a specific concentration of aqueous hormone solution. Phytohormones, which are organic substances that are naturally produced by plants, play a crucial role in regulating plant growth and development, even in microdoses. The most significant outcomes are observed when the seeds undergo phytohormone priming under stress conditions. For example, exogenous GA-treated maize seeds exhibited a significantly enhanced photosynthetic rate in seedlings and improved the final kernel quality; GA treatment also increased antioxidant enzyme activity and alleviated oxidative stress, resulting in improved maize growth under cold or salt stress [114,115,116]. While ABA has proven effective in enhancing tolerance to alkaline stress [117], seeds subjected to IAA priming showed increased vigor and better germination [118]. Seed priming with CTKs imparted salt stress in wheat [119] and drought tolerance in soybean plants [120]. Maize seeds primed with BRs enhanced the germination index, root length, and photosynthetic rate under drought stress [121]. Wheat seeds primed with salicylic acid resulted in an increased germination percentage and reduced the time of germination in both saline and non-saline conditions [122]. 

### 3.6. Seed Priming and Stress Condition

Currently, studies on seed priming predominantly focus on stress experiments. It has been found that the use of endogenous metabolites, such as dopamine, salicylic acid, and proline, can effectively enhance the stress resistance of the post-germination stage [123,124,125,126,127,128,129]. This efficacy is primarily attributed to the increase in soluble sugar and free proline content in the plants following treatment, which aid in maintaining osmotic balance in seedlings during stress and inhibiting membrane lipid peroxidation reactions [127,128,129]. Tomato seedlings developed from seeds treated with a citric acid solution exhibited enhanced phosphorus absorption under low-level phosphorus stress [130]. Substances such as melatonin, choline chloride, calcium chloride, and potassium chloride can also serve as seed priming agents to enhance the antioxidant capacity [128,131,132]. 

Halopriming and osmo-priming are also seed priming technologies which can assist in coping with stress conditions. Halopriming, seed priming with salt, can enhance plant growth and decrease saline intolerance under salt stress conditions during the subsequent seedling stage [133]. Halopriming with KNO_3_ enhanced the germination index, final germination percentage, root length, shoot length, and seedling fresh weight in tomato plants [134]. Germination and seedling growth are enhanced under salt and drought stress when sunflower seeds are primed with KNO_3_ and water [135]. Osmo-priming is a commonly adopted priming technique and offers a highly attractive solution for improving seed germination performance and crop stand establishment [136]. Osmo-priming with PEG significantly impacted the germination index, germination percentage, and seed vigor in soybean plants [137]. 

Seed priming technology has also proven to enhance crop yield [138,139,140]. The faba bean seeds with hydropriming exhibited a higher seed yield (12.0%) [138]. Nutrient priming with zinc under zinc-deficient soil conditions increased the final yield of bread wheat by 27.1% [139]. Hormonal priming treatments significantly enhanced the wheat yield across all genotypes under both normal and drought-stress conditions [140]. Research into seed priming has evolved over the last five decades, exploring a spectrum of priming methods that extend beyond the chemical methods.

Overall, seed priming can break seed dormancy, and enhance seed germination, seedling uniformity, plant performance, and stress resistance. It may be postulated that the positive effect observed is due to the priming treatment inducing activities related to germination and causing abiotic stress to the seed, which results in the formation of a “priming memory” within the seed, facilitating the transition of dry seeds to the germinated state, leading to an increase in germination potential, and also mediating greater stress resistance in germinating primed seeds when they encounter stress [136]. However, the positive effects of priming tend to diminish over time, and the longevity of primed seeds decreases during storage, which impose limitations on its application. Notably, the primed seeds exhibit significant deterioration at room temperature, with relative air humidity being a key factor influencing the sprouting performance; a high relative air humidity accelerates this process [141]. One possible explanation for this phenomenon could be that the activation of the seed cell cycle during the priming treatment is not completely halted by the subsequent back-drying operation, leading to inevitable cellular senescence. Conversely, cell cycle inhibitors have been demonstrated to prevent seed deterioration, resulting in improved storability following the priming treatments [142]. Moreover, the effect of salinity, used in halopriming and chemical priming, is very complex. Salinity will provide specific nutrient ions and cause ionic stress and osmotic stress will result in the formation of the “priming memory”, but the redundant accumulation of ions such as Na^+^ in primed seeds will also give rise to ionic toxicity, which will cause secondary stress, namely damage to cell structures and macromolecules [143]. This toxicity may be caused by using exogenous agents that influence the internal physiological state of the seed, rather than by direct involvement in the seed’s metabolic pathways. Furthermore, different effects on different plants are still observed despite the use of endogenous phytohormones for seed treatment; for example, only rice, but not tobacco, maize, wheat, or soybean plants, showed a significant increase in the submerged germination of salicylic acid-treated seeds [144]. This indicates that screening for more diverse and effective endogenous agents for priming is of great important.

## 4. Conclusions with Future Perspectives

Seed dormancy and germination, essential aspects of agronomy, are regulated by various regulatory pathways, highlighting the need for a comprehensive understanding of the underlying mechanisms and factors influencing them. Throughout seed dormancy and germination, along with the accompanying oxidative reactions, increased levels of reactive oxygen species can lead to the carbonylation of proteins, resulting in the loss of functions of modified proteins [145]. However, it is worth noting that the accumulation of reactive oxygen species also serves as a crucial signaling mechanism, particularly in controlling cellular activities during the post-ripening stage of seeds [146]. The dynamic equilibrium of ABA and GA contents in plant organisms, coupled with species-specific sensitivity, significantly affects the physiological and biochemical processes of seed dormancy and germination. 

The network of the physiological and biochemical metabolic processes within the seeds during germination is highly complex. As the exploration of seed germination mechanisms continues, future research should incorporate diverse biological technologies, such as transcriptomics, metabolomics, and proteomics, and integrate various seed types to deepen our understanding of seed germination mechanisms and elucidate the underlying patterns. For example, the internal storage material of seeds contains a variety of compounds, and different seeds may display different germination performance due to the difference in the content of a certain compound. Detecting such differences and employing seed priming technologies to validate the efficacy of the seed treatment offers a valuable approach to unraveling the mechanisms governing seed dormancy and germination, as well as a means of screening for valuable priming substances. At the same time, in acknowledging the limitations inherent in seed priming techniques, it is crucial to identify strategies to overcome these shortcomings and improve these technologies. Collectively, these efforts aim to make seed priming technologies an effective means of rescuing and protecting endangered germplasm, thus ensuring the genetic stability of germplasm for the long-term preservation of valuable genetic resources.

## Data Availability

Data availability is not applicable to this article as no new data were created or analyzed in this study.
